# Karnofsky Performance Scale and Neurological Assessment of Neuro-Oncology Scale as Early Predictor in Glioma

**DOI:** 10.31557/APJCP.2020.21.11.3387

**Published:** 2020-11

**Authors:** Pricilla Yani Gunawan, Andi Asadul Islam, Julius July, Ilhamjaya Patellongi, Muhammad Nasrum, Tiara Aninditha

**Affiliations:** 1 *Department of Neurology, Faculty of Medicine, Universitas Pelita Harapan, Jl. M.H.Thamrin Boulevard 1100, Lippo Village, Tangerang 15811, Indonesia. *; 2 *Department of Neurosurgery, Faculty of Medicine, Universitas Hasanuddin, Jl.Perintis Kemerdekaan KM 10, Makassar, Indonesia. *; 3 *Department of Neurosurgery, Faculty of Medicine, Universitas Pelita Harapan, Jl. M.H.Thamrin Boulevard 1100, Lippo Village, Tangerang 15811, Indonesia. *; 4 *Department of Physiology, Faculty of Medicine, Universitas Hasanuddin, Jl.Perintis Kemerdekaan KM 10, Makassar, Indonesia. *; 5 *Molecular Biology and Immunology Laboratory, Faculty of Medicine, Universitas Hasanuddin, Jl.Perintis Kemerdekaan KM 10, Makassar, Indonesia.*; 6 ^*6*^ *Department of Neurology, Faculty of Medicine, Universitas Indonesia. Jl. Diponegoro No.71, Jakarta, Indonesia. *

**Keywords:** Functional scale, KPS, NANO scale, glioma

## Abstract

**Objective:**

Glioma is one of the most frequent and disabling primary brain tumour. Patients are not only dealing with survival, but also quality of life, which remains another major concern. Karnofsky Performance Scale (KPS) is one of the most commonly used scale to assess patients’ quality of life. A recent scale, known as Neurological Assessment of Neuro-Oncology Scale, has surfaced to examine neurological disability caused by brain tumour. Previous study showed this scale to be superior to KPS in predicting survival. However, these scales have never been used to foresee functional scale improvement during disease progression. We sought to determine whether initial KPS and NANO Scale can predict functional scale improvement 2 months after surgery.

**Methods:**

Patients with glioma grade II-IV were included in the study. IDH mutation and MGMT methylation were tested. KPS and NANO scale were examined before surgery and 2 months after surgery. Favorable outcome (FO) was defined as improvement in functional scale 2 months after surgery. Patients initial functional scales were analyzed towards favorable outcome.

**Results:**

Glioma WHO grade II, III and IV was found in 17 patients (36.2%), 3 patients (6.4%) and 27 patients (57.4%) respectively. Median KPS before and 2 months after surgery were 50 (30-80) and 60 (0-100), whereas median NANO scale before and 2 months after surgery were 5 (0-12) and 3 (0-12). Favorable outcome was found in 63.8% (KPS) and 78.7% (NANO Scale). Patients initial functional scales were significantly related to FO.

**Conclusion:**

Good initial functional scales are 4 to 5 times likely of having a favorable outcome 2 months after surgery.

## Introduction

Primary brain tumour remains one of the most disabling and lethal disease. Data from International Agency for Research on Cancer 2018 shows incidence of primary brain and CNS tumour worldwide of approximately 296,851. At the same year, The Global Cancer Atlas showed that 5,354 people in Indonesia were diagnosed with primary brain tumour. Glioma represents 27% of all tumours and 80% of all malignant tumours (Ostrom et al., 2015). Glioblastoma accounts for the majority of gliomas (55,1%), and combined glioblastoma and astrocytomas account for about 75% of all gliomas.

Glioblastoma is an incurable tumour, and poor prognosis continue to prevail despite substantial treatment (Gorlia et al., 2012). In literature, age of patients, performance status, IDH (isocitrate dehydrogenase) mutation, MGMT (O6 Methylguanine-DNA methyltransferase) methylation status, and treatment were commonly known as predictive factors for survival in glioblastoma. Nonetheless, therapeutic intervention itself has been shown to decrease patients’ quality of life. Randomised Clinical Trials have pointed out that radiation therapy may prolong the time to recurrence, but on the other hand may decrease patients’ quality of life and cognitive functions (Aaronsen et al., 2011; Correa et al., 2008; Mainio et al., 2006).

In assessing quality of life and performance status, the Karnofsky Performance Scale (KPS) were generally used. In 2017, a new Neuro-Oncology Scale named the Neurological Assessment of Neuro-Oncology (NANO) Scale was developed in hope to assess patients with brain tumour more objectively, taking into account neurological manifestations while predicting prognosis (Rano et al., 2017). These performance scales were functioned as a scale parameter in assessing patients’ performance pre-treatment, to analyse their overall survival. It was found that performance status estimated by the NANO scale was significantly associated with overall survival, and showed to be a more powerful method to predict the prognosis of glioblastoma than the KPS, during both initial diagnosis and disease progression (Lee et al., 2018). However, not many studies assessed the fluctuations in functional scale during the progression of the disease, nor used a functional scale improvement as a favourable outcome. 

In this study, we sought to investigate whether initial KPS and NANO Scale can serve as an early predictor in functional scale improvement 2 months after surgery. Other possible predictors were also analysed towards functional scale improvement. 

## Materials and Methods

*Patient selection*

All patients with histologically confirmed glioma grade II-IV who present at 3 major hospitals from June 2019 to June 2020 were consented to be included in the study. Patient underwent standardized therapy (surgery and radiotherapy with or without chemotherapy) and tumor tissue was analyzed to determine IDH-1 mutational status and methylation of MGMT. Functional scale was assessed using KPS and NANO scale before surgery and re-assessed 2 months after. Favorable outcome (FO) was defined as: 1) increment of KPS by 10 or more calculated from difference of KPS 2 months after surgery and initial KPS; and 2) decrement of NANO Scale by 1 or more, calculated from difference of initial NANO and NANO_2_ months after surgery. This study was approved by Medical Ethical Research Committee, Universitas Hasanuddin, No: 1232/UN4.6.4.5.31/PP36/2019.

*Molecular analysis*

*IDH Mutation testing*

To determine the IDH-1 mutational status, we used high resolution melting (HRM) analysis and direct sequencing. Genomic DNA was extracted from paraffin-embedded tumor tissues using the QIAampRDNAMicroKit (QIAGEN) according to the manufacturers’ protocol. IDH1 alterations of the mutational hotspot codons R132 were assessed by HRM analysis and direct sequencing of PCR-amplified fragments, which were generated during the HRM procedure with the PCR primers. Primers used were: IDH1-forward 5’-CGGTCTTCAGAGAAGCCATT-3’ and IDH1-reverse 5’-GCAAAATCACATTATTGCCAAC-3’. Samples with conflicting findings by HRM and direct sequencing were re-tested and only HRM-positive samples confirmed by direct sequencing were considered mutated.

*MGMT Methylation testing*

Genomic DNA was extracted using the QIAampRDNAMicroKit (QIAGEN). DNA underwent bisulfite treatment to convert all unmethylated cytosine to uracil while leaving 5-methylcytosine unaltered. It was then eluted in DNase-free water. Methylation analysis was carried out using Real Time based and Methylation Specific PCR (MSP). Primers used were: MGMT-forward 5’TTTCGACGTTCGTAGGTTTTCGC-3” and MGMT-reverse 5”-GCACTCTTCCGAAAACGAAACG-3’. Classification was carried out binary, MGMT unmethylated and MGMT methylated.

*Statistical analysis*

Correlations between functional scales before and 2 months after surgery were analyzed using Pearson correlation test. To identify cut off value of KPS and NANO scale, ROC curve analysis was done. Potential prognostic variables including age (≥45 versus <45 years), gender, IDH mutational status, MGMT methylation, and preoperative functional scale were analyzed toward FO using Chi-Square test. Multivariate analysis was conducted using logistic regression. All calculations were performed using statistical analysis software package, IBM SPSS Statistics version 24. A probability value of <0.05 was considered statistically significant. 

## Results

As much as 47 patients were enrolled in this study. Mean age was 43.79 (±16.43) years old, with predominantly male (61.7%). Most tumors were located at frontal lobe, and the most common presenting symptom was headache. Most patients underwent surgery, radiation, and chemotherapy. Glioma WHO grade II was found in 17 patients (36.2%), grade III in 3 patients (6.4%) and grade IV in 27 patients (57.4%) as depicted in Table 1.

IDH mutant R132H (IDH-1 mutation) was found in 15 patients (31.9%). It was found in 58.8% of glioma grade II, 33.3% of glioma grade III, and 14.8% of glioma grade IV. MGMT methylation was found in 33 patients (70.2%). It was seen in 82.4% of glioma grade II, 66.7% of glioma grade III, and 63% of glioma grade IV. In patients with IDH-mutant, as much as 86.7% have MGMT methylation as well. Coexistence of MGMT methylation and IDH mutation was seen in 47.1% glioma grade II, 33.3% glioma grade III, and 14.8% glioma grade IV (Table 2). 

**Figure 1:**
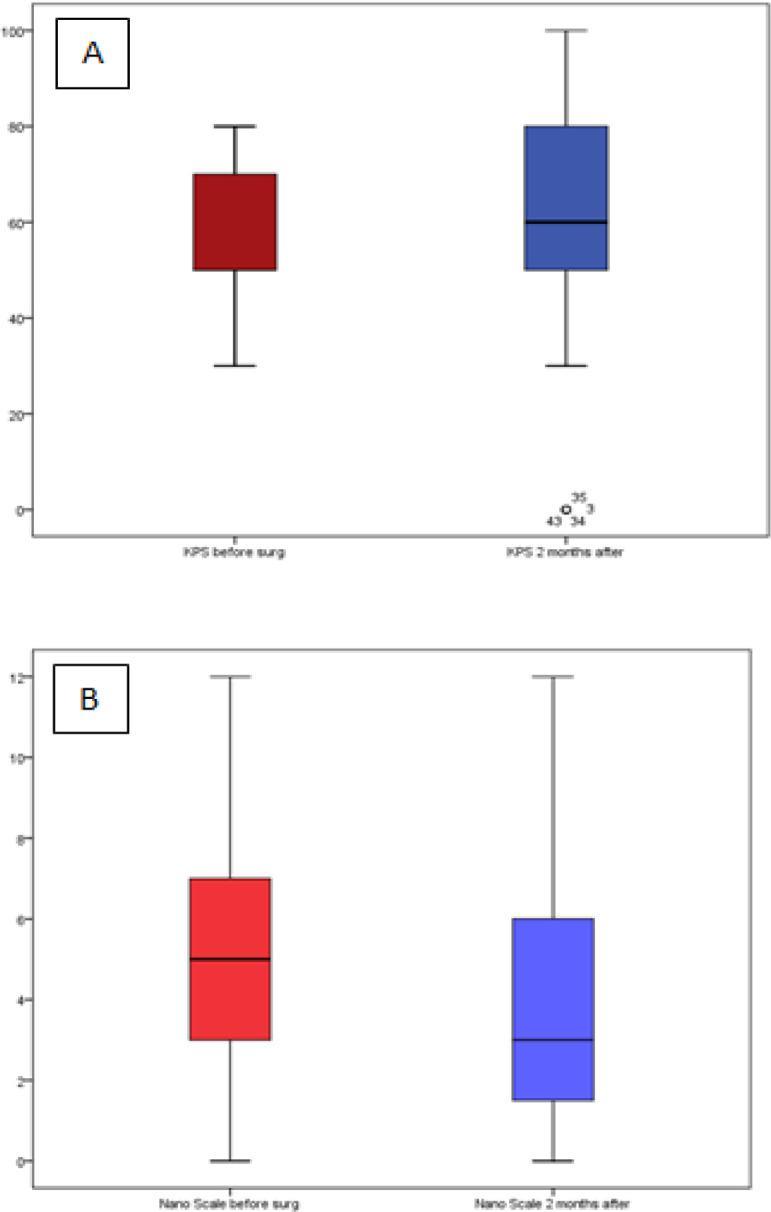
Functional Status Assessed Using (A) KPS and (B) NANO scale. (Red box: before surgery, Blue box: 2 months after surgery)

Functional status before and 2 months after surgery were depicted in Figure 1. Median KPS before and 2 months after surgery were 50 (30-80) and 60 (0-100), whereas median NANO scale before and after surgery were 5 (0-12) and 3 (0-12). Favorable outcome KPS and NANO Scale was found in 63.8% and 78.7% respectively. In patients with IDH mutant, 80% had FO KPS, and 100% had FO NANO. Median KPS and NANO before surgery according to IDH mutation and MGMT methylation were depicted in Table 3. 

**Figure 2:**
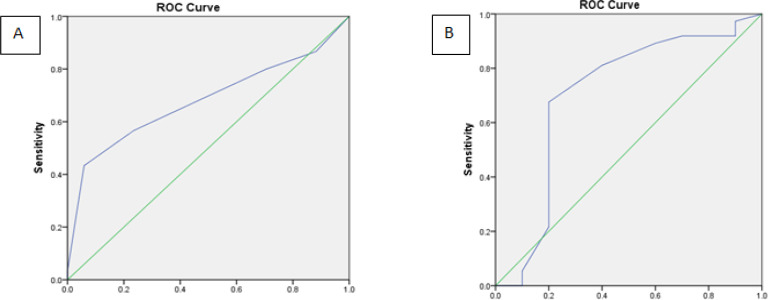
ROC Curve Analysis for (A) KPS and (B) NANO scale before surgery towards favorable outcome

Pearson correlation test showed significant correlation between functional scales before surgery and 2 months after (KPS r=0.771; p=0.000 vs NANO r=0.945; p=0.000). ROC curve analysis to determine the cut off value of KPS and NANO scale before surgery towards favorable outcome were portrayed in Figure 2. Cut off value along with its sensitivity and specificity was outlined in Table 4. Using the cut off value, KPS and NANO scale before surgery as well as IDH mutation and MGMT methylation status were analyzed towards FO. Bivariate (Table 5 and 6) and multivariate analysis showed KPS (p=0.033; OR=4.25; 95%CI 1.12-16.12) and NANO Scale (p=0.047; OR=5.1; 95%CI 1.02–25.54) before surgery were statistically significant towards FO.

**Table 1:** Baseline Characteristics

Variable	N (%)
Age(years)	
≥45	25 (53.2)
<45	22 (46.8)
Gender	
Male	29 (61.7)
Female	18 (38.3)
Tumour location	
Frontal	30 (63.8)
Temporal	10 (21.3)
Parietal	4 (8.5)
Occipital	3 (6.4)
Symptoms	
Headache	40 (85.1)
Seizure	23 (48.9)
Loss of consciousness	10 (21.3)
Hemiparesis/Hemiplegic	31 (65.9)
Cognitive disturbance	6 (12.8)
Language disturbance	23 (48.9)
Therapy	
Surgery and Radiation	6 (12.8)
Surgery, Radiation and Chemotherapy	41 (87.2)
Histopathological diagnosis	
WHO grade II	
Diffuse Astrocytoma, IDH-mutant	5 (10.6)
Diffuse Astrocytoma, IDH-wild type	3 (6.4)
Oligoastrocytoma, IDH-mutant	2 (4.3)
Oligodendroglioma, IDH-mutant	1 (2.1)
Oligodendroglioma, IDH-wildtype	2 (4.3)
Gemistocytic astrocytoma, IDH-mutant	2 (4.3)
Gemistocytic astrocytoma, IDH-wild-type	1 (2.1)
Pleomorphic xanthoastrocytoma	1 (2.1)
WHO grade III	
Anaplastic oligodendroglioma, IDH-mutant	1 (2.1)
Anaplastic xanthoastrocytoma	2 (4.3)
WHO grade IV	
Glioblastoma, IDH-mutant	4 (8.5)
Glioblastoma, IDH-wildtype	21 (44.7)
Small cell glioblastoma, IDH-wildtype	1 (2.1)
Giant cell glioblastoma, IDH-wildtype	1 (2.1)

**Table 2:** Distribution of Glioma in Accordance with MGMT Methylation and IDH Mutation Status

MGMT methylation status	IDH Mutation status	WHO grade		
		II	III	IV
Methylated	Mutant	8	1	4
	Wild type	6	1	13
Unmethylated	Mutant	2	0	0
	Wild type	1	1	10

MGMT methylation status refers to the MGMT gene promoter methylation, which may be methylated or unmethylated. IDH mutation status is the mutational status of the isocitrate dehydrogenase 1 gene (codon R132), which may be mutant (refers to the presence of somatic mutations) or wild type (without somatic mutations). MGMT: O6-Methylguanine-DNA methyltransferase; IDH: isocitrate dehydrogenase

**Table 3:** Functional Scales According to IDH1 Mutation and MGMT Methylation

Biomarker	Functional scale before surgery	
	KPS	NANO
IDH mutation		
Mutant	60 (30-70)	4 (2-11)
Wildtype	50 (30-80)	6 (0-12)
MGMT methylation		
Methylated	50 (30-80)	4 (1-12)
Unmethylated	50 (30-70)	6 (0-12)

IDH, isocitrate dehydrogenase; MGMT, O6-Methylguanine-DNA methyltransferase

**Table 4:** ROC Curve Analysis of Functional Scales before Surgery towards Favorable Outcome

Functional Scale	Cut off	Sensitivity (%)	Specificity (%)	LR +	LR -	AUC
KPS	55	56.7	76.5	2.41	0.57	0.680
NANO	6.5	81.1	60	2.03	0.32	0.705

**Table 5:** Predictive Factors and Favorable Outcome using KPS

Variable		Favorable outcome KPS		OR	95% CI	P value
		+	-			
Age (years)						
	<45	16	6	2.1	0.62-7.14	0.234
	≥45	14	11			
IDH mutation						
	Mutant	12	3	3.11	0.73-13.2	0.114
	Wildtype	18	14			
MGMT methylation						
	Methylated	22	11	1.5	0.42-5.41	0.534
	Unmethylated	8	6			
KPS						
	60-100	17	4	4.25	1.12-16.12	0.028
	0-50	13	13			

**Table 6:** Predictive Factors and Favorable Outcome Using NANO Scale

Variable		Favorable outcome NANO		OR	95% CI	P value
		+	-			
Age (years)						
	<45	18	4	1.42	0.34-5.88	0.73
	≥45	19	6			
IDH mutation						
	Mutant	15	0	*	*	0.019
	Wildtype	22	10			
MGMT methylation						
	Methylated	27	6	1.8	0.42-7.74	0.456
	Unmethylated	10	4			
NANO Scale						
	0-6	30	4	6.43	1.42-29.08	0.017
	7-12	7	6			

*, Not calculated due to a cell frequency equal to zero

## Discussion

Besides overall survival, functional status of glioma patients has been one of the concerns in choosing and commencing with treatment options. Functional scale after radiotherapy was one of the many concerns, since quality of life was shown to decreased significantly after radiation (Aaronsen et al., 2011; Correa et al., 2008; Mainio et al., 2006). However, increase in functional scale after surgery and during chemoradiotherapy has never been specifically studied. 

Unlike many previous studies that only included patients with high or low functional scale, we included all patients with glioma, disregard of their functional scale on admission. Filtering only patients with high or low scale was deemed unnecessary, since the measured outcome was increase in functional status, which is the difference between pre surgery and 2 months after surgery scale. Nonetheless, we analyzed all glioma patients (low and high grade), instead of confining certain grade, in hope of gaining a conclusion on which scale that may predict favorable outcome and could be applied to all glioma patients. 

Mean age of patients in this study was 43.79 (±16.43) years old. This is relatively younger than age with the highest glioma incidence commonly studied, which is between 45 and 60 years old (Altieri et al., 2014; El-Zein et al., 2005). In this study, glioblastomas accounted for more than half (57.4%) of all gliomas, followed by diffuse astrocytoma (17%) and oligodendrogliomas (8.5%). This histopathological distribution is similar to CBTRUS Statistical Report 2011-2015 (Ostrom et al., 2018).

Gliomas, as one of the most common malignant primary brain tumors, have been subject to the greatest investigations, including biomarker research. IDH mutation, have been associated with improved prognosis in glioma (Myung et al., 2012; Songtao et al., 2012) . These mutations are common in grade II and III gliomas, but rare in glioblastoma (Yan et al., 2009). In this study, we found a comparable result where IDH mutations were most common in glioma grade II and III (58.8% and 33.3% respectively). 

Another biomarker commonly accepted for its predictive and prognostic value is MGMT gene promoter methylation. Previous studies show MGMT methylation was found mostly in high-grade tumors (Bady et al., 2016; Majchrzak-Celińska et al., 2015). In this study, MGMT methylation was mostly seen in glioma grade II. This might be due to several possibilities. First, the fact that MGMT methylation coexist with IDH mutation, which is commonly seen in low grade gliomas, made their occurrence much more frequent than high grade gliomas with less IDH mutation. Second, studies across the world has shown race difference in clinical value of MGMT methylation. Survival benefit observed in Caucasians without TMZ treatment, was not distinctly perceived in Asians (Zhao et al., 2018). Hence, it is possible that proportion of MGMT methylation itself differs between tumor grade in different race. Third, the use of proper methodology for the detection of DNA methylation is crucial. In this study we used MSP analysis, which is the appropriate, recommended, and proven to be a preferred method (Majchrzak-Celińska et al., 2015; Switzeny et al., 2016). However, MSP do not detect low, partial, or mosaic DNA methylation (which is still questionable whether this pattern shows any significant utility). Hence, the difference in testing method could reveal a distinct distribution of methylation across tumor grading.

Coexistence of MGMT methylation and IDH mutation was seen in 86.7%. It was less frequent in glioma grade IV (14.8%) with comparing result to a previous study (Molenaar et al., 2014). It was commonly accepted that glioblastomas with IDH mutation and MGMT methylation harbors the best survival (Molenaar et al., 2014; Yang et al., 2015). Owing to their more frequent coexistence in low grade gliomas, it is sensible that low grade gliomas have better survival.

Functional status before and 2 months after surgery using KPS and NANO scale shows improvement. We obtain a cut off for KPS and NANO towards FO. Cut off 55 in KPS (which divides KPS as high 60-100 and low 0-500 has the sensitivity of 56.7% and specificity of 76.5%. Whereas NANO scale with cut off 6.5 (which categorize NANO scale as low 0-6 and high 7-23) has the sensitivity of 81.1% and specificity of 60%. These findings differ from previous study (Lee et al., 2018). For high KPS they obtain a range of 80-100, whereas for NANO Scale low score is 0-7. Cut off as well as sensitivity and specificity were different with our findings due to different study outcome which is overall survival.

The relationship between IDH mutation and MGMT methylation toward FO was not statistically significant (except for IDH mutation towards FO NANO Scale); however, as shown in Table 5 and 6, the tendency towards positive FO in IDH mutant and MGMT methylated tumors was observed. Both biomarkers have previously been shown to significantly correlate with better survival but have never been analyzed towards improvement in short term follow up. 

The initial functional scale significantly correlates with favorable outcome. High initial KPS and low initial NANO scale are 4 and 5 times likely to have a FO 2 months after surgery. This finding outlined the importance of assessing KPS and NANO scale on admission to predict FO. KPS before surgery can be a predictor of increased quality of life 2 months after surgery, and NANO scale before surgery can predict improvement of neurological deficit 2 months after surgery. However, compared to KPS, NANO scale possess a stronger correlation towards favorable outcome (NANO r=0.945 vs KPS r=0.771), and a higher AUC and sensitivity. Thus, it is ascertained that NANO scale is superior to KPS in predicting favorable outcome 2 months after surgery. 

We did a follow up using 2 scales for several reasons. First, KPS is commonly used in previous trials to categorize patients and examine its survival. NANO Scale is a relatively new scale shown to be a better predictor (compared to KPS) for overall survival. Nonetheless, these scales have never been compared to short term follow up in terms of patients’ improvement. Second, both scales have distinct items of evaluation. KPS evaluate patients’ quality of life and capability in commencing work/tasks. NANO Scale focuses on neurological deficits which is simplified and easy to assess. Thus, it is more scrupulous to see these scales as complementing each other. Patients quality of life does not necessarily be determined only by their ability to perform daily tasks, but also to be able to walk properly, communicate with family and have an intact visual and sensory function. 
